# Polygenic score for Alzheimer’s disease identifies differential atrophy in hippocampal subfield volumes

**DOI:** 10.1371/journal.pone.0270795

**Published:** 2022-07-13

**Authors:** Balaji Kannappan, Tamil Iniyan Gunasekaran, Jan te Nijenhuis, Muthu Gopal, Deepika Velusami, Gugan Kothandan, Kun Ho Lee, for the Alzheimer’s Disease Neuroimaging Initiative

**Affiliations:** 1 Gwangju Alzheimer’s & Related Dementia Cohort Research Center, Chosun University, Gwangju, Republic of Korea; 2 Department of Biomedical Science, Chosun University, Gwangju, Republic of Korea; 3 Health Systems Research & MRHRU, ICMR-National Institute of Epidemiology, Tirunelveli, Tamil Nadu, India; 4 Department of Physiology, Sri Manakula Vinayagar Medical College and Hospital, Puducherry, Tamil Nadu, India; 5 Biopolymer Modeling and Protein Chemistry Laboratory, Centre of Advanced Study in Crystallography and Biophysics, University of Madras, Chennai, Tamil Nadu, India; 6 Korea Brain Research Institute, Daegu, Republic of Korea; Nathan S Kline Institute, UNITED STATES

## Abstract

Hippocampal subfield atrophy is a prime structural change in the brain, associated with cognitive aging and neurodegenerative diseases such as Alzheimer’s disease. Recent developments in genome-wide association studies (GWAS) have identified genetic loci that characterize the risk of hippocampal volume loss based on the processes of normal and abnormal aging. Polygenic risk scores are the genetic proxies mimicking the genetic role of the pre-existing vulnerabilities of the underlying mechanisms influencing these changes. Discriminating the genetic predispositions of hippocampal subfield atrophy between cognitive aging and neurodegenerative diseases will be helpful in understanding the disease etiology. In this study, we evaluated the polygenic risk of Alzheimer’s disease (AD PGRS) for hippocampal subfield atrophy in 1,086 individuals (319 cognitively normal (CN), 591 mild cognitively impaired (MCI), and 176 Alzheimer’s disease dementia (ADD)). Our results showed a stronger association of AD PGRS effect on the left hemisphere than on the right hemisphere for all the hippocampal subfield volumes in a mixed clinical population (CN+MCI+ADD). The subfields CA1, CA4, hippocampal tail, subiculum, presubiculum, molecular layer, GC-ML-DG, and HATA showed stronger AD PGRS associations with the MCI+ADD group than with the CN group. The subfields CA3, parasubiculum, and fimbria showed moderately higher AD PGRS associations with the MCI+ADD group than with the CN group. Our findings suggest that the eight subfield regions, which were strongly associated with AD PGRS are likely involved in the early stage ADD and a specific focus on the left hemisphere could enhance the early prediction of ADD.

## Introduction

Alzheimer’s disease dementia (ADD) is a debilitating neurodegenerative disease and the most common form of dementia that currently affects millions around the world and its occurrence is expected to triple by 2050 [1]. Early prognosis and diagnosis are crucial in optimal disease management and intervention. Brain imaging and genetic data have played a vital role in forming the diagnostic criteria. Brain imaging studies have shown the medial temporal lobe regions to be highly vulnerable to aging and AD. Neuroimaging studies have well-established the atrophy in medial temporal regions [2, 3], especially in the hippocampus associated with aging [4] and AD [5, 6].

Hippocampal volume is among the widely-studied and well-established criteria for the early diagnosis of AD [7]. The hippocampus can be divided into sub-regions with distinct functional characterizations [8] and recent developments in neuroimaging techniques have made it possible to obtain proxy subfield information to study the heterogeneous regions of the hippocampus in greater detail [9]. Studies have been performed on these subfields to determine the earliest affected regions for AD, aging, and other neurological disorders. Previous studies on AD have narrowed down the early atrophy initiation site around the CA1-subiculum regions and expect the atrophy to be moving inward-out with the progression of the disease [10–12]. Moreover, some studies reported asymmetric hippocampal atrophy among AD patients. Interestingly, some studies suggested AD patients are highly susceptible to substantial level of asymmetric atrophy in hippocampal subfield volumes [13–16].

Hippocampal subfield volumes are shown to have a high heritability rate and thus can be used as quantitative phenotypes in genetic association studies [17]. Heritability is a measure of the degree of variation in a trait due to the genetic variations among the individuals in a population. The understanding of the genetic influence on various underlying mechanisms specific to neurological processes of different subfields will help in the prognosis and diagnosis.

Genome-wide association studies (GWASs) investigate the association of genetic variants mainly of individual single-nucleotide polymorphisms (SNPs) on complex traits under study. Numerous such genetic variants are said to have diverse and complex etiologies and synergistic effects on many complex traits. Identification of each genetic variant is important to understand the risk prediction, disease risk, and population differences of the gene toward a trait. GWAS on AD cases and normal controls have identified many genetic variants associated with diseases. For instance, genes like *APOE*, *TREM2*, *CLU*, *CR1*, and many others with functional roles in pathways related to brain function, suggesting high-risk associations with AD [18–20], have been reported using GWAS. Studies have shown high heritability of around 60–80% associated with late-onset AD [21]. However, the individual effects of the reported genetic risk loci are smaller in comparison to the combined polygenic effect. An individual SNP has a modest effect and explains a small proportion of the risk. The synergetic effect of multiple risk loci and each with their own minor risk leads to a higher AD incidence in most cases [22, 23].

The increasing use of GWAS and the subsequent need for analyzing the polygenic effects of the SNPs have led to the wide use of approaches like polygenic risk score (PGRS) analysis using different methods. The PGRS is a single value estimate based on the genetic architecture of an individual expressing the genetic liability towards a trait or a disease of interest. PGRS is the cumulative genetic risk of a phenotype of interest in an individual based on the genetic variants in the genome.

However, the effect of the PGRS on the individual subfields of the hippocampus remains unclear. In the present study, we, investigate the relationship between the hippocampal subfields and the PGRS. We calculated and examined 1) the PGRS effect for individual hippocampal subfields and their hemispheric lateralization or asymmetry in the total population independent of their clinical status, and 2) the differences in the PGRS effect for individual groups classified based on their clinical status. We expect 1) strong AD PGRS associations of CA1 and subiculum regions with the MCI and ADD groups and less strong associations with the CN group, 2) weak AD PGRS association in the CA3, parasubiculum, and fimbria with the MCI and ADD groups, and stronger associations with the CN group, being the farthest regions from the AD-related atrophy initiation site, and 3) strong AD PGRS association with the left hemisphere volumes and less strong associations with the right hemisphere volumes.

## Methods

### Study participants

Data used in the preparation of this article were obtained from the Alzheimer’s disease Neuroimaging Initiative (ADNI) database (http://adni.loni.usc.edu). The present study includes data from 1086 participants; 319 cognitively normal control elderly, 591 participants with mild cognitive impairments, and 176 patients diagnosed with ADD. The eligibility criteria for all the ADNI subjects can be found at https://adni.loni.usc.edu/methods/documents/. ADNI used the National Institute of Neurological and Communicative Disorders and Stroke/Alzheimer’s Disease and Related Disorders Association (NINCDS-ADRDA) criteria for the clinical diagnosis of probable AD. A summary of the participant’s demographic characteristics is shown in Table 1.

**Table 1:** Demographic characteristics of the study population.

	CN	MCI	AD
Number of subjects (n)	319	591	176
Age^a^^,^^b^	75.21±5.27	73.43±7.43	75.40±7.76
Male (%)^c^	47.33	39.08	44.31
Level of education (years)^a^^,^^d^	16.34±2.67	15.91±2.85	15.00±3.04
MMSE^a^^,^^e^	29.08±1.12	27.64±1.77	23.32±2.04

Values are expressed as mean ± standard deviation (SD).

CN, cognitive normal; MCI, mild cognitively impaired; AD, Alzheimer’s disease dementia; MMSE, Mini-Mental State Examination.

^a^The *P*-values were calculated using the general linear model; Bonferroni post hoc test was also performed when F-test was significant.

^b^Main interaction among groups: *F*_*2*, *1080*_ *= 11*.*51*, *p = 1*.*10E-5*. (*Age*). Post hoc: CN versus MCI, 4.10E-5; MCI versus AD, 5.96E-3; CN versus AD, 1.00.

^c^The *P*-value were calculated using the *χ*^*2*^ *test*: *χ*^*2*^ *= 6*.*10*, *p = 0*.*04*. *(Gender)*

^d^Main interaction among groups: *F*_*2*, *1080*_ *= 14*.*30*, *p = 7*.*35E-7*. Post hoc: CN versus MCI, 5.15E-3; MCI versus AD, 4.05E-3; CN versus AD, 4.11E-7. *(Education)*

^e^Main interaction among groups: *F*_*2*, *1080*_ *= 680*.*56*, *p = 6*.*39E-192*. Post hoc: CN versus MCI, 1.86E-33; MCI versus AD, 7.01E-142; CN versus AD, 1.11E-189. (MMSE)

### MRI acquisition and data processing

**Fig 1:**
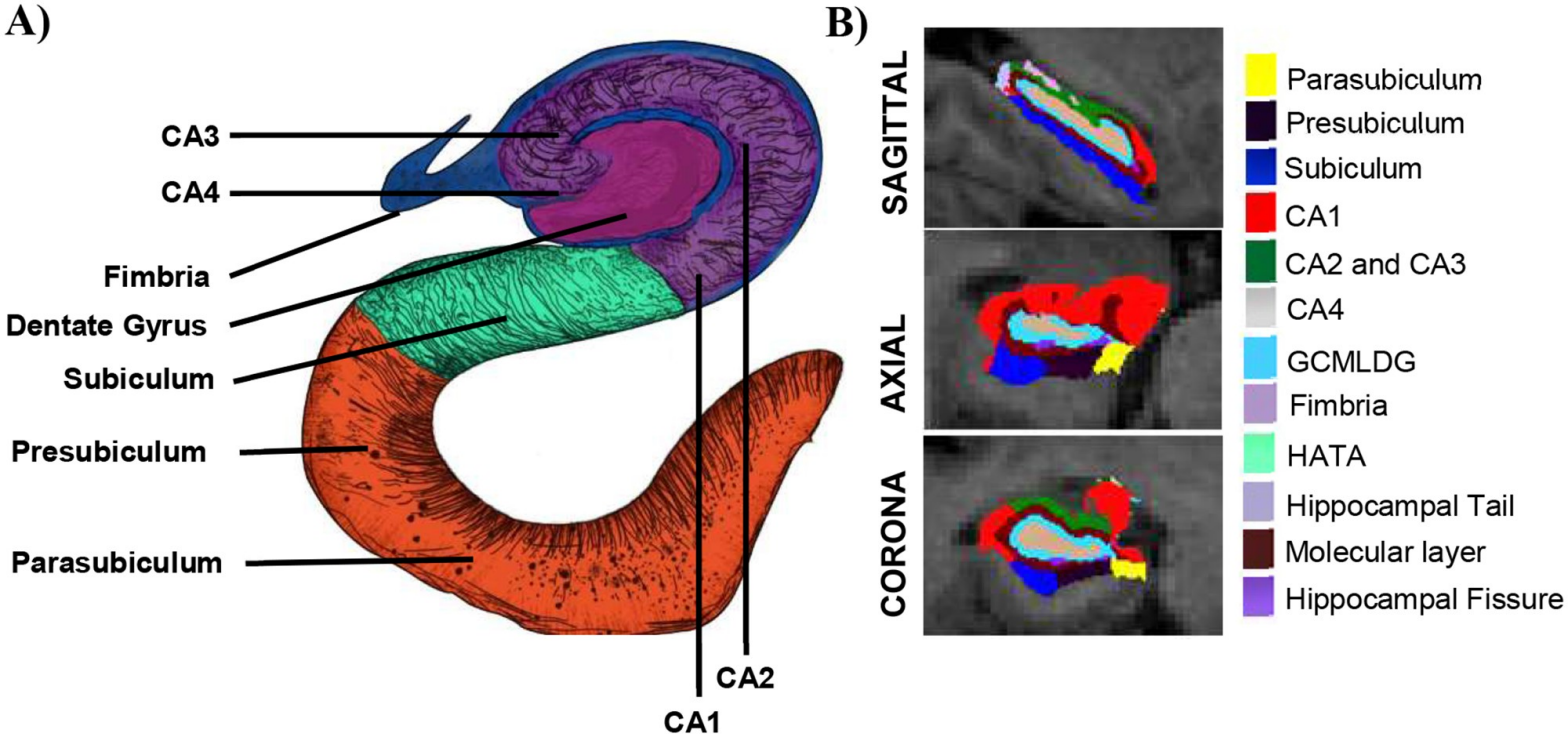
Hippocampal subfields as segmented using the Freesurfer. **A)** A transverse section of hippocampus displaying the different regions of the hippocampus, **B)** An illustration of the heterogenous hippocampus segmented into 12 subfields using an automated segmentation technique used in this study based on an atlas developed using Bayesian inference algorithm. CA—cornu ammonis, GCMLDG—granule cell layer of the dentate gyrus, HATA—hippocampus-amygdala-transition area.

Cross-sectional T1-weighted structural MRI scans with raw data acquisition matrix of 192 × 192 × 166, voxel size of 1.25 × 1.25 × 1.2 mm^3^, repetition time/echo time of 8020/50 ms, 0.4 × 0.4 × 2.0 mm^3^ resolution, minimum 24 slices, and acquisition time: 8.1 mins were used. Further details on ADNI imaging protocols can be found at http://adni.loni.usc.edu/methods/documents/mriprotocols/. The T1 images were processed using Freesurfer v6.0 (https://surfer.nmr.mgh.harvard.edu/fswiki) software and extracted whole hippocampus volume and the hippocampal subfield volumes. Detailed documentation of the procedures can be found elsewhere [24–26]. We used an updated automated technique for the reliable segmentation of the hippocampal subfields using ultra-high-resolution T1 MRI data recently incorporated into the Freesurfer software suite [9]. The hippocampus was segmented into the whole hippocampus, hippocampal-amygdala-transition-area (HATA), hippocampal tail (tail), subiculum (SUB), presubiculum (PSUB), parasubiculum (ParaSUB), cornu ammonis 1(CA1), cornu ammonis 3 (CA3), cornu ammonis 4 (CA4), fimbria, granule cells in the molecular layer of the dentate gyrus (GC-ML-DG), molecular layer HP (ML), and hippocampal fissure (fissure) as shown in Fig 1. For statistical analysis, we used intracranial volumes (ICV) as covariates. Since we had access to the ICV values for all the study subjects from our previous study [27], we used ICV values estimated from FreeSurfer v5.30 (https://surfer.nmr.mgh.harvard.edu/fswiki) to reduce computational time. We note that the two versions of the software package show only very little differences between the ICV values estimated [28]. Since there was no substantial changes in the hippocampal subfield segmentation tool used in the FreeSurfer version 7.1 compared to version 6.0 [29], we used FreeSurfer v6.0 for estimating hippocampal subfield volumes. In addition, the amygdala segmentation was highly focused in the FreeSufer v7.1 [30] and this suggests that using FreeSurfer v7.1 makes no difference to the values estimated from the hippocampal subfield segmentation algorithm implemented in the FreeSurfer v6.0 [29].

### SNP genotype data

SNP genotype data from ADNI-1, ADNI-GO, and ADNI-2 cohorts were downloaded from the ADNI LONI database (http://adni.loni.usc.edu/). Genotype data from the whole genome sequencing (WGS) project genotyped with Illumina Omni 2.5M Chip (Illumina, Inc, San Diego, CA, USA), ADNI-1 project genotyped using Illumina HumanOmniExpress Bead Chip (Illumina, Inc, San Diego, CA, USA) and ADNI-GO/2 project genotyped with Illumina Human610-Quad Bead Chip (Illumina, Inc, San Diego, CA, USA) were considered in this study. Subjects from ADNI-3 were not included as the data for ADNI-3 was recently added. The quality control was performed separately for these genotype datasets. Individuals with call rate <95%, gender inconsistencies, heterozygosity rate (± 3 *SD* from the mean) were excluded. SNPs with call rate <95%, minor allele frequency (MAF) <1%, Hardy-Weinberg equilibrium (HWE) test *P*-value <10^−6^ were excluded. After quality control, imputation was performed for all datasets with a pre-phased reference panel using the European Ancestry population implemented in the Haplotype Reference Consortium (HRC) panel version 1.1. After imputation, low-quality SNPs with info score <0.5 and MAF <0.01 were excluded.

For the analysis, subjects who underwent MRI scanning and DNA genotyped were included. 727 non-Hispanic white subjects primarily enrolled in the ADNI whole-genome sequencing (WGS) project, 355 subjects that were not enrolled in the ADNI WGS project, but enrolled in the ADNI-1 project were included. Additionally, 4 subjects that were not present in both WGS and ADNI-1 projects but enrolled in the ADNI-GO/2 project were included. All the genotype datasets were merged for the analysis, which finally comprised 1,086 subjects and 7,485,124 SNPs.

### Statistical analysis

All statistical analyses for the demographic variables were performed using IBM SPSS Statistics (Version 26.0. Armonk, NY: IBM Corp.). All analyses entailed two-tailed significance testing and controlled for the covariates age, sex, years of education, and estimated total intracranial volume. One-way analysis of variance and Bonferroni post hoc correction for multiple comparisons was used for continuous demographic variables, and chi-squared tests were performed for categorical demographic variables. We considered *P*-values <0.05 as significant.

Linear regression on 12 hippocampal subfield volumes and whole hippocampus volume involving 7,485,124 SNPs were performed using PLINK [31] software. Principal components (PCs) accounting for the population substructure were calculated with the Smartpca program using EIGENSOFT [32]. Tracy-Widom statistics were used to identify the significantly associated PCs with both 12 hippocampal volumes and whole hippocampus volume. Linear regression was performed with age, sex, field strength, intracranial volume, education, and 3 PCs as covariates.

### Estimation of Polygenic Risk Scores (PGRS)

PGRSs for 12 hippocampal subfield volumes and whole hippocampus volume were estimated using the profile option in the PLINK software. Summary statistics data downloaded from the International Genomics of Alzheimer’s Project (IGAP) consortium (https://www.niagads.org/) study conducted by Lambert et al., [33] were included as training data. The IGAP study comprises 37,154 control subjects and 17,008 AD cases. The data were linkage disequilibrium (LD) clumped with clump function (—clump) available in PLINK software by excluding SNPs located within 500 kilobases and having *r*^2^ > 0.25 with a significantly-associated SNP. The analysis was performed by iterating over a range of *P*-values (GWAS significance level or polygenic threshold *P* < 5 × 10^−8^, *P* < 1 × 10^−7^, *P* < 1 × 10^−6^, *P* < 1 × 10^−5^, *P* < 1 × 10^−4^, *P* < 1 × 10^−3^, *P* < 0.01, *P* < 0.05, *P* < 0.1, *P* < 0.1, *P* < 0.3, *P* < 0.5) to determine the best-fit *P*-value threshold.

## Results

### Demographics

A total of 1086 participants—319 cognitively normal, 591 participants with mild cognitively impaired, and 176 patients diagnosed with ADD—were analyzed. Age (*F*_*2*, *1080*_ *= 11*.*51*, *p = 1*.*10E-5*), gender (*χ*^*2*^ *test*: *χ*^*2*^ *= 6*.*10*, *p = 0*.*04*), level of education (*F*_*2*, *1080*_ *= 14*.*30*, *p = 7*.*35E-7*), and MMSE (*F*_*2*, *1080*_ *= 680*.*56*, *p = 6*.*39E-192*) were all significantly different between the diagnostic groups (Table 1).

### AD PGRS effect on bilateral hippocampal subfields

**Fig 2:**
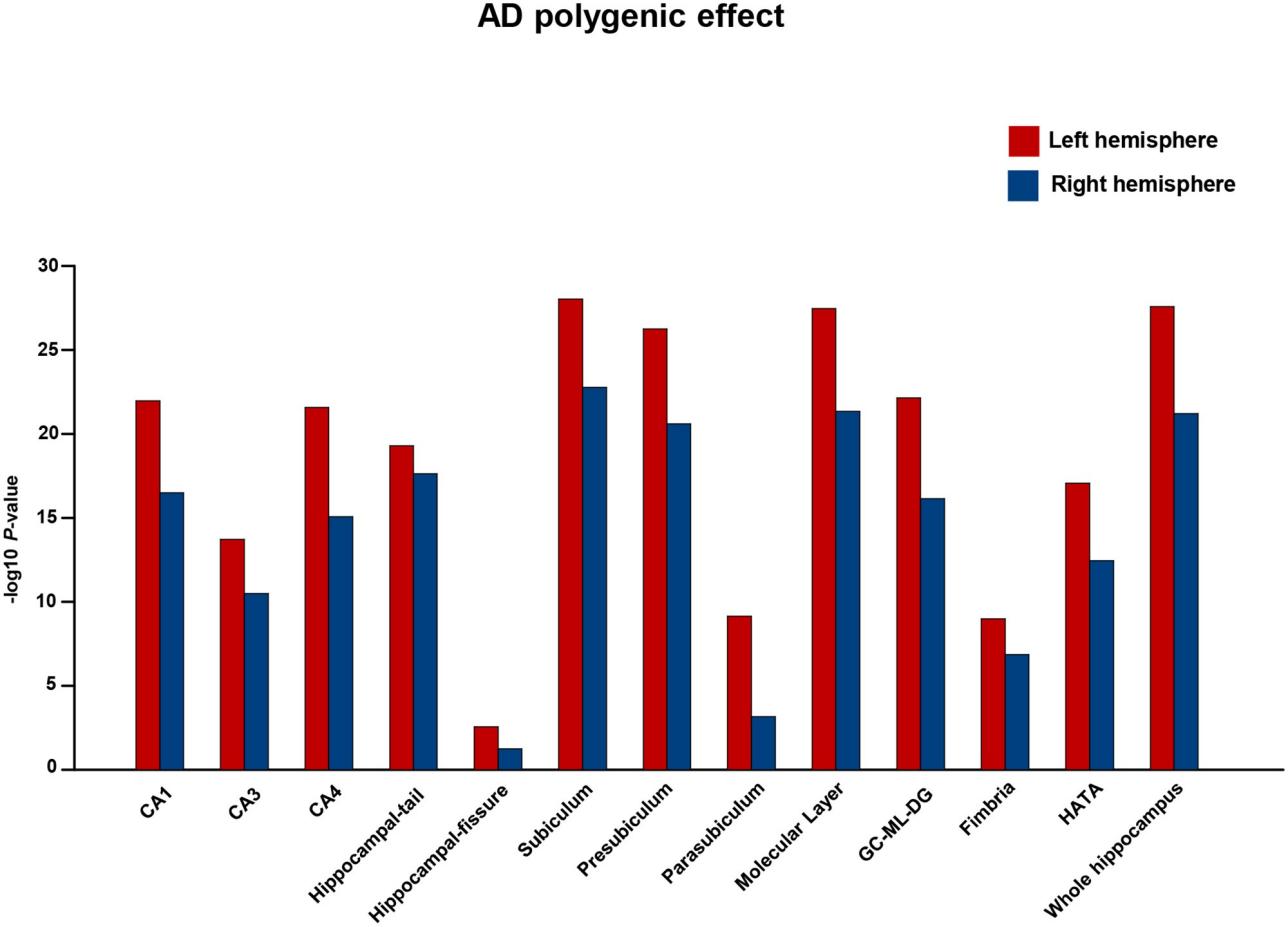
Hippocampal subfields exhibit asymmetric hemispherical effects for AD PGRS. AD polygenic analysis on hippocampal subfields showed asymmetric hemispherical differences. Best fit AD polygenic risk score correlation *P*-values were shown for each hippocampal subfield. The x-axis shows hippocampal subfields and the y-axis shows–log10 *P*-values from the best-fit AD polygenic risk score correlations. The red-colored bar plot indicates the left hemisphere and the blue color indicates the right hemisphere.

In this analysis, the mixed clinical status population (CN+MCI+ADD) was included to evaluate the hemispherical differences. AD polygenic association analysis was performed on the left and right hemispheres of the 12 hippocampal subfields and the whole hippocampus to test for hemispherical differences. The results showed a strong AD PGRS effect on the left hemisphere of the 12 subfields and the whole hippocampus than the right hemisphere of 12 subfields and the whole hippocampus (S1 Table and Fig 2).

### AD PGRS effect on hippocampal subfields of clinically diagnosed group

**Fig 3:**
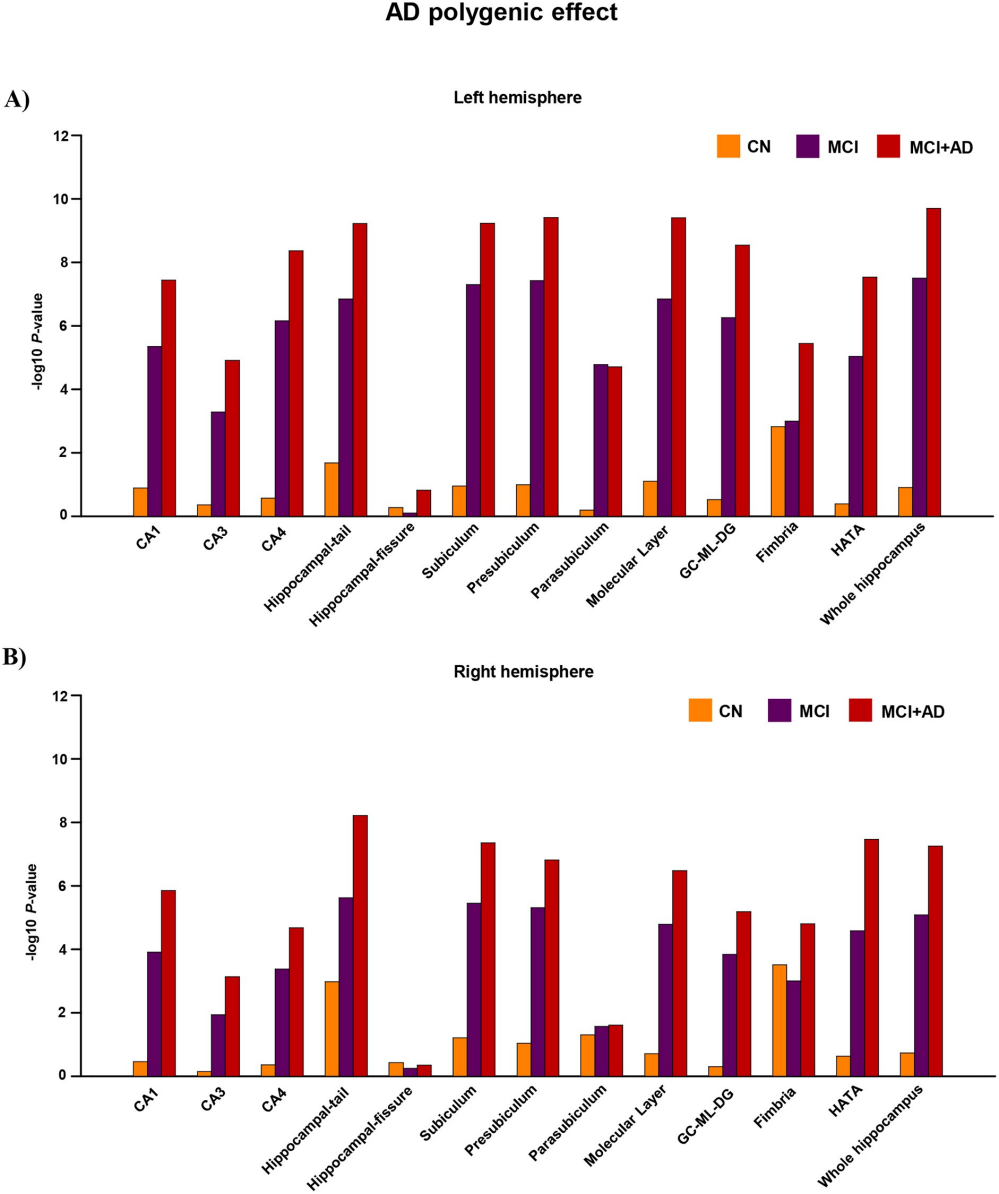
Hippocampal subfields in MCI and AD subjects show differential AD PGRS effects. MCI and AD subjects exhibit differential AD polygenic effects for the hippocampal subfields. Best-fit AD polygenic risk score correlation *P*-values were shown for each hippocampal subfield stratified by cognitively normal (CN), mild cognitively impaired (MCI), and Alzheimer’s disease (AD) groups. Due to the low sample size for the AD group, MCI subjects were combined with AD subjects. The x-axis shows hippocampal subfields stratified for CN, MCI, and MCI+AD. The y-axis shows–log10 *P*-values from the best-fit AD polygenic risk score correlations. The orange-colored bar plot indicates CN, purple color indicates MCI, and red color indicates MCI+AD. **A)** The top bar plot represents the left hemisphere and the plot **B)** below represents the right hemisphere.

The AD PGRS effect was evaluated in the clinically diagnosed groups stratified as CN, MCI, and MCI+ADD). Due to a small number of ADD subjects (*n* = 176), we combined MCI and ADD (MCI+ADD) subjects to evaluate the AD polygenic effect among the ADD group. The analyses were performed on the left and right hemispheres of the hippocampal subfields and the whole hippocampus. All the 12 hippocampal subfields showed a stronger AD PGRS effect on the left hemisphere than the right hemisphere in CN, MCI, and MCI+ADD groups (S2 Table and Fig 3). Likewise, the whole hippocampus exhibited stronger AD PGRS effect on the left hemisphere (*P*_T_<0.01, *r*^*2*^ = 0.04, *P* = 1.93×10^−10^) than on the right hemisphere (*P*_T_<5×10^−8^, *r*^*2*^ = 0.03, *P* = 5.54×10^−8^) in all groups. We hypothesized that the MCI+ADD group can demonstrate the AD PGRS effect much better than CN and MCI groups, so we focused more on evaluating the MCI+ADD group and compared it with the CN group.

In the left hemisphere of MCI+ADD group, subfields like CA1 (*P*_T_<0.01, *r*^*2*^ = 0.03, *P* = 3.51×10^−8^), CA4 (*P*_T_<0.01, *r*^*2*^ = 0.03, *P* = 4.23×10^−9^), hippocampal-tail (*P*_T_<1×10^−4^, *r*^*2*^ = 0.04, *P* = 5.89×10^−10^), subiculum (*P*_T_<0.01, *r*^*2*^ = 0.04, *P* = 5.77×10^−10^), presubiculum (*P*_T_<0.01, *r*^*2*^ = 0.04, *P* = 3.84×10^−10^), molecular layer (*P*_T_<0.01, *r*^*2*^ = 0.04, *P* = 3.91×10^−10^), GC-ML-DG (*P*_T_<0.01, *r*^*2*^ = 0.03, *P* = 2.79×10^−9^) and HATA (*P*_T_<0.01, *r*^*2*^ = 0.03, *P* = 2.91×10^−8^) showed strong association for AD polygenic risk related atrophy than CN group. While, CA3 (*P*_T_<0.01, *r*^*2*^ = 0.02, *P* = 1.20×10^−5^), parasubiculum (*P*_T_<0.01, *r*^*2*^ = 0.02, *P* = 1.95×10^−5^), fimbria (*P*_T_<0.01, *r*^*2*^ = 0.02, *P* = 3.56×10^−6^) showed moderate association for AD polygenic-risk-related atrophy. Hippocampal fissure (*P*_T_<0.001, *r*^*2*^ = 0.003, *P* = 0.15) showed no association (*P*<0.05) for the AD polygenic-risk-related atrophy.

In the right hemisphere of MCI+ADD group, parasubiculum (*P*_T_<1×10^−7^, *r*^*2*^ = 0.006, *P* = 0.02) showed a weaker association for the AD polygenic risk effect than the left hemisphere. Moreover, parasubiculum in the right hemisphere showed minor difference in the strength of their association for AD polygenic effect among CN (*P*_T_<0.01, *r*^*2*^ = 0.01, *P* = 0.05), MCI (*P*_T_<0.01, *r*^*2*^ = 0.008, *P* = 0.03) and MCI+ADD (*P*_T_<1×10^−7^, *r*^*2*^ = 0.006, *P* = 0.02) groups. Likewise, fimbria showed little differences in the strength of their association for AD polygenic effect among CN (*P*_T_<0.01, *r*^*2*^ = 0.03, *P* = 3.0×10^−4^), MCI (*P*_T_<0.01, *r*^*2*^ = 0.01, *P* = 9.8×10^−4^) and MCI+ADD (*P*_T_<0.01, *r*^*2*^ = 0.02, *P* = 1.56×10^−5^). Similar to the left hemisphere, hippocampal fissure (*P*_T_<5×10^−8^, *r*^*2*^ = 0.001, *P* = 0.44) in the right hemisphere of MCI+ADD group showed no association (*P*<0.05) for the AD polygenic risk related atrophy.

A Bonferroni-adjusted significance threshold of *p = 0*.*05/25 structures/3 groups = 6*.*67E-4* was used.

## Discussion

In the present study, we aimed to understand the associations among polygenic risk for Alzheimer’s disease, volumes of individual hippocampal subfield, hemispheric asymmetry or lateralization, and diagnostic group variations among cognitive normal, mild cognitively impaired, and Alzheimer’s disease dementia individuals. To the best of our knowledge, the current study is the first to investigate the hemispherical asymmetry or lateralization and the differences in the association of AD PGRS on hippocampal subfield volumes along the AD spectrum. Multiple comparison issues were corrected using the stringent post hoc Bonferroni correction.

Our results showed differences in the strength of AD PGRS association on left and right hippocampal volumes of the individual clinical diagnostic groups (CN, MCI and ADD) and the mixed clinical status population (CN+MCI+ADD). The left hemisphere showed a greater inclination to neurodegeneration than the right, predominantly. The subfields CA1, CA4, hippocampal tail, subiculum, presubiculum, molecular layer, GC-ML-DG, and HATA showed stronger PGRS associations in the MCI+ADD than in the CN. In contrast, the subfields CA3, parasubiculum, and fimbria showed moderate AD PGRS associations in the MCI+ADD than CN. There was no AD PGRS association for the hippocampal fissure between the groups.

Earlier studies on the association between AD PGRS and hippocampal volumes report a clear genetic influence on total hippocampal volume [34], and a high AD PGRS effect on the total left hippocampal volume [35]. In line with the previous study, our findings showed a strong association for the AD PGRS effect on the left hippocampal subfield volumes. A previous study involving young participants reported an association of AD PGRS with CA1 and fissure regions [36]. Although previous studies primarily focused on investigating the association of AD PGRS on hippocampal volume or subfield volumes only among the young population, our study evaluated the AD PGRS effect on the hippocampal subfields along the AD continuum.

Aging, neurodegenerative and certain neuropsychiatric conditions generally display hemispherical asymmetry in their atrophy patterns. Structural brain changes are observed across the lifespan of adults [37] including the later stages of aging [38]. Previous studies showed asymmetric hippocampal atrophy between CN, MCI, and ADD [39, 40]; specifically, the left hemisphere is much more vulnerable to degeneration than the right hemisphere [41, 42]. Generally, the left hemisphere is engulfed much faster than the right during the AD-mediated degeneration. However, the right hemisphere follows the similar degeneration with a time lag. This leads due to the asymmetrical brain atrophy among AD patients [42]. The significant atrophy in the left hemisphere is may be due to increased β-amyloid deposition in the left hemisphere of AD patients [43], specifically, increased β-amyloid deposition in the left precuneus/cuneus and medial-temporal (fusiform, parahippocampal and entorhinal), an adjacent region to the hippocampus causes increased atrophy in these regions [44]. It should be noted that the atrophy starts in the entorhinal cortex and then spreads to the hippocampus [45]. The occurrence of this pathomechanism suggests that the increased beta-amyloid deposition probably leads to the increased atrophy in the left hippocampal subfield volumes. In line with the previous studies, our study involving a group with mixed clinical status has demonstrated greater atrophy due to AD PGRS effect in the left hemisphere of all subfield regions than the right hemisphere (except for subfields; hippocampal fissure, and parasubiculum, bilaterally) and this strong AD PGRS effect on the left hemisphere was also observed in the whole hippocampus volume. Thus, considering the subregions in the two hemispheres as individual entities would be appropriate to explain the hemispheric functional lateralization.

Hippocampal atrophy is among the strongest predictors of AD progression, as the volumetric measure and the atrophy rates are shown to be efficient in the classification of CN, MCI, and ADD [6]. Hippocampal subfield regions reported having high heritability among healthy young, adult, and elderly groups [46]. Moreover, understanding the association of AD PGRS with hippocampal subfields will help in focusing on specific subfield regions in the early prediction and preventive interventions.

Like the mixed clinical population, the left hemisphere showed a stronger association for AD PGRS related atrophy than the right hemisphere among CN, MCI, and MCI+ADD groups, respectively. In the CN group, other than fimbria, the rest of the regions did not show any significant AD PGRS association with hippocampal subfield volumes, bilaterally. We found a stronger association of AD PGRS with CA1, CA4, hippocampus-tail, subiculum, presubiculum, molecular layer, GC-ML-DG, and HATA regions among the MCI group, which is considered as an intermediate stage for ADD [47]. Moreover, this association was much stronger in the left hemisphere. Further, our results suggest that atrophy in the CA1, CA4, hippocampus-tail, subiculum, presubiculum, molecular layer, GC-ML-DG, and HATA regions could initiate in the early stages of ADD, specifically in the left hemisphere.

AD PGRS models are seen to have high predictive accuracy in classifying MCI converters into late-onset AD [48]. Since the number of subjects in the AD group was inadequate to capture the AD PGRS effect, we combined the MCI and ADD groups into the (MCI+ADD) group to be able to evaluate the AD PGRS effect in ADD subjects. However, like the results in the MCI group, CA1, CA4, hippocampus-tail, subiculum, presubiculum, molecular layer, GC-ML-DG, and HATA regions showed a stronger association with AD PGRS in the MCI+ADD group than in the CN group. This suggests that these eight subfield regions are strongly associated with AD-mediated atrophy in the early and late stages of the disease. As anticipated, the strength of association in these eight subfield regions among MCI+ADD was greater than in the MCI group and this is due to the presence of ADD subjects in the group. So, further tests of an AD PGRS effect in the subfield regions among the ADD group require additional, larger sample sizes.

Age-related volumetric atrophy was absent in fimbria, CA1, subiculum, presubiculum, hippocampal tail, molecular layer, and HATA, but was seen in CA2/3, CA4, and dentate gyrus [49]. However, AD-related atrophy was found in CA1, CA2/3, CA4, subiculum, parasubiculum, presubiculum, and dentate gyrus [10, 12, 50–53].

Based on the AD PGRS risk, the trajectory of influence is early and high on the left hemisphere [54]. Carlesimo et al. suggested the subfield volume change initiates around the subiculum and presubiculum [12]. Subsequently, the change might travel toward the outward regions and might finally reach the CA3, fimbria, and parasubiculum, the farthest regions from the initiation site [11]. In line with a previous study, our results showed a strong AD PGRS effect in subiculum and presubiculum along with CA1, CA4, hippocampal tail, molecular layer, GC-ML-DG, and HATA. This suggests that these eight subfield regions are likely associated with early-stage AD-related atrophy. The moderate association with AD PGRS observed in CA3, parasubiculum, and fimbria suggests that these regions are likely involved in the later stages of AD-related atrophy.

The present study has various salient features. First, we used a very large training dataset from the IGAP consortium for a robust and precise AD PGRS modeling. Second, we evaluated the AD PGRS effect using 1,086 subjects, which included subjects along the AD continuum aged between 55 and 90, unlike previous studies that predominantly focused on young adults aged less than 55. However, the number of subjects in the AD group was inadequate to capture the AD PGRS effect, so we combined MCI and ADD (MCI+ADD) group to substantially evaluate the AD PGRS effect among ADD subjects, which is a clear limitation of this study.

Though, recent studies on the hippocampal subfield volumes used high resolution T2-weighted images [55, 56], we selected T1-weighted images for two main reasons. First, several previous studies conducted their hippocampal subfield automated segmentation using T1-weighted images, which may be helpful for us to validate our findings with previous studies due to commonality in the type of neuroimaging. Second, we want to use the most commonly available T1-weighted images so that our study results may be incorporated into a future meta-analysis on the subfield volumes. Our results must be interpreted cautiously as our study did not use T2-weighted high resolution images and this adds to the limitation of our study.

In sum, the current study suggests that there is a stronger AD PGRS association with the left hemisphere than with the right hemisphere. We also found differential AD PGRS associations for certain hippocampal subfields. Most importantly, the eight subfield regions with strong AD PGRS association highlighted in the ADD group are likely associated with early stages of ADD. Taken together, our study suggests that focusing on eight subfield regions specifically on the left hemisphere could help predict ADD at its early stages.

## Supporting information

S1 Table Polygenic risk scores of the hippocampal subfields for the left hemisphere and the right hemisphere in the mixed clinical status population. (XLSX)

S2 Table Polygenic risk scores of the hippocampal subfields for the left hemisphere and the right hemisphere in the cognitively normal, mild cognitively impaired, Alzheimer’s disease dementia, and the mild cognitively impaired + Alzheimer’s disease dementia groups. (XLSX)
